# Evaluation of incomplete maternal smoking data using machine learning algorithms: a study from the Medical Birth Registry of Norway

**DOI:** 10.1186/s12884-020-03384-y

**Published:** 2020-11-23

**Authors:** Liv Grøtvedt, Grace M. Egeland, Liv Grimstvedt Kvalvik, Christian Madsen

**Affiliations:** 1 Department of Health and Inequality, Norwegian Institute of Public Health, Sandakerveien 24c, Bygg B, 0473 Oslo, Norway; 2 Department of Health Registry Research and Development, Norwegian Institute of Public Health, Bergen, Norway; 3 Department of Global Public Health and Primary Care, University of Bergen, Bergen, Norway; 4 Department of Biomedicine, University of Bergen, Bergen, Norway

**Keywords:** Pregnancy, Smoking, Hospitals, Ethnic groups, Education, Birth weight, Machine learning, Informed consent

## Abstract

**Background:**

The Medical Birth Registry of Norway (MBRN) provides national coverage of all births. While retrieval of most of the information in the birth records is mandatory, mothers may refrain to provide information on her smoking status. The proportion of women with unknown smoking status varied greatly over time, between hospitals, and by demographic groups. We investigated if incomplete data on smoking in the MBRN may have contributed to a biased smoking prevalence.

**Methods:**

In a study population of all 904,982 viable and singleton births during 1999–2014, we investigated main predictor variables influencing the unknown smoking status of the mothers’ using linear multivariable regression. Thereafter, we applied machine learning to predict annual smoking prevalence (95% CI) in the same group of unknown smoking status, assuming missing-not-at-random.

**Results:**

Overall, the proportion of women with unknown smoking status was 14.4%. Compared to the Nordic country region of origin, women from Europe outside the Nordic region had 15% (95% CI 12–17%) increased adjusted risk to have unknown smoking status. Correspondingly, the increased risks for women from Asia was 17% (95% CI 15–19%) and Africa 26% (95% CI 23–29%). The most important machine learning prediction variables regarding maternal smoking were education, ethnic background, marital status and birth weight. We estimated a change from the annual observed smoking prevalence among the women with known smoking status in the range of − 5.5 to 1.1% when combining observed and predicted smoking prevalence.

**Conclusion:**

The predicted total smoking prevalence was only marginally modified compared to the observed prevalence in the group with known smoking status. This implies that MBRN-data may be trusted for health surveillance and research.

**Supplementary Information:**

**Supplementary information** accompanies this paper at 10.1186/s12884-020-03384-y.

## Background

The Medical Birth Registry of Norway (MBRN) has since 1999 registered self-reported maternal smoking status at the beginning and end of pregnancy with marked declines noted between 1999 and 2014 [1, 2]. The decline in smoking prevalence was found in all demographic and educational groups, and in 2017 Norway had a smoking prevalence of 4% at the beginning (first trimester) and 2% at the end of pregnancy (third trimester) [2, 3].

Among many health effects, smoking has been consistently related to intrauterine growth restriction measured as small-for-gestational age (SGA). Further, smoking cessation is known to improve fetal growth [4]. The validity of smoking data from MBRN is important, as misclassification would bias estimates of smoking prevalence and the association between smoking and adverse pregnancy outcomes.

In accordance with the current MBRN-regulations from 1/1–2002; §1–7, mothers are given an option to refrain from providing smoking information. Written information about the right to refrain from reporting smoking are provided to the mother in Norwegian and English. Data about consent or non-consent are included in the notification forms at the hospitals before being sent to MBRN [5].

The quality of the smoking data from the MBRN has been assessed earlier [5]. Briefly, the proportion of births with unknown maternal smoking status has varied over time, between geographical regions, hospitals, and by mothers’ country of birth. During the years 2005–2014, electronic birth records were gradually introduced, with a corresponding reduction in incomplete smoking data [5]. Electronic records may give fewer opportunities to ignore required information. Problems of using smoking information from years with a highly varying unknown category of smoking, as well as the need to omit large numbers of births with incomplete data in research studies have been described elsewhere [2, 6]. Information about the magnitude of possible errors due to incomplete data on smoking during pregnancy would facilitate appropriate use of the data in future research and public health surveillance.

The current study evaluates whether incomplete data on smoking in the MBRN contributed to a biased smoking prevalence among pregnant women in first trimester using a traditional and a machine learning approach. First, we investigated how the available demographic and administrative data predicted the tendency not to provide smoking information. Secondly, we used machine learning to model the relationship between the variables for mothers with known smoking status, in order to predict the prevalence of smoking for mothers with unknown smoking status. The extent of potential bias introduced by assuming that the smoking information to some degree was missing-not-at-random (MNAR) was evaluated by comparing the observed and the predicted smoking prevalence. The main aim of the present study was to investigate if the high and varying proportion of unknown smoking status among pregnant women introduced biased estimates of smoking prevalence, which in turn could negatively affect the validity of the MBRN-data.

## Methods

The MBRN is a mandatory nationwide population-based registry of all births. Starting at the first antenatal visit, an obstetric nurse midwife or physician fills in an antenatal chart with demographic, reproductive and lifestyle data that follows the woman throughout her pregnancy. Maternal smoking status is registered by checkboxes as non-smoker, occasional smoker (less frequent than daily), or daily smoker. For daily smokers the number of daily smoked cigarettes is recorded [7, 8]. At delivery, information from the antenatal chart is transferred to the MBRN notification form. Most of the antenatal information is mandatory, but for registration of smoking status, the mothers may refrain from providing information. The MBRN is used for surveillance and research, but not in medical treatment. When the woman refrains to give smoking information, we define this as *“unknown smoking status” as* opposed to *“known smoking status”* which includes smokers and non-smokers.

The MBRN data was linked by the unique personal identification number assigned to every resident of Norway to the population register for the information on country of birth, immigration category (born in Norway or abroad with Norwegian or immigrant parents etc.) and to the education database in Statistics Norway. Women born in Norway, or those with one or both parents born in Norway, were defined as “Norwegian”.

The educational scales are regularly harmonised with the International Standard Classification of Education (last update 2011). Women in our study population from the Nordic countries had educational data with less than 2% missing, compared to 22, 32, and 28% missing among women from non-Nordic European countries, Africa, and Asia, respectively.

Maternal age, parity, marital status, educational level, and small-for-gestational age are variables known to be associated with maternal smoking and misclassification of smoking status [2, 4, 9–12]. Therefore, we evaluated these aforementioned variables as they related to “known” and “unknown” smoking status. Small-for-gestational age (SGA) was used as a proxy for growth inhibition and its prevalence was used to help evaluate whether smoking was randomly missing [4, 11]. We included country region of origin, type of notification form (paper-based vs electronic) and hospitals in the multivariable analyses, as we had reason to believe that the rates of incomplete smoking data varied within these subdivisions [5]. We stratified the bivariable analyses into two periods “1999–2006” and “2007–2014” due to decreasing smoking prevalence, increasing proportion of women from outside the Nordic countries [2], introduction of electronic notification forms in the latter time period, and, in spite of decreasing smoking rates, decreasing mean birth weights of the newborn [3, 13].

We assumed that information on maternal smoking in the unknown group could be missing for various reasons. The information in some cases may be missing-at-random (MAR) and dependent on available information such as the mother’s age or educational level, while in others may be MNAR and dependent on the true smoking status of the mothers. In order to estimate annual scenarios of MNAR for our population in the unknown group, we used the ratio of SGA for the groups of unknown and known smoking status, to scale the observed smoking prevalence. SGA-ratio was applied, as smoking may be seen as an important reason for MNAR among pregnant women [4, 14].

### Study population

The MBRN data set (1999–2014) initially comprised 960,408 birth records among 556,006 mothers. We excluded non-viable births and multiple births resulting in 919,584 births. Non-viable births were defined as births with weight below 500 g and gestational age below 22 weeks. The consent-variable had available information on smoking status for 904,982 births, while this information was missing for 1.6% of births (Supplement S1).

The unit of analyses was births, where each woman could have contributed to the data more than once during the observation period.

### Description of variables

*Smoking* was, unless otherwise stated, defined as both daily and occasionally smoking (combined) in first trimester of pregnancy.

*Mothers’ consent* to provide smoking information was coded 0 for “consent” and 1 for “non-consent”.

*Year of childbirth* was treated as a continuous variable in addition to being dichotomised as “period 1 (1999-2006)” and “period 2 (2007-2014)”.

*SGA-10* was defined as a birth weight < 10th percentile by sex and gestational age based upon Norwegian standards [11].

*Maternal age* was continuous, and/or grouped into four categories (< 19; 20–29; 30–39; 40 years and above). *Parity* was continuous, and/or classified as (no previous birth; one previous birth; and two or more previous births).

*Marital status* was classified as “married/ cohabitant”, or as “single” combining unmarried, single, divorced, separated, widowed and others.

*The highest achieved educational level* for each woman at the year of childbirth was grouped as “high education”; any college or university education ≥14 years of schooling; “medium education”; high school 11–13 years of schooling; and “primary education”; ≤10 years of schooling.

*Country region of origin* was categorized into: “Nordic” (Norwegian, Swedish, Danish, Finnish or Icelandic); “Non-Nordic Europeans”; “African”; “Asian”; and “other” (North- and South America and Oceania).

*Notification of births* sent from the hospitals to MBRN were classified as either “paper forms” or “electronic forms”.

*The institution level (hospitals)* made it possible to separate the 16 largest hospitals, and all hospitals smaller than 20,000 births during all years were collapsed into one reference group.

In addition, the variables *birth weight in quartiles, sex* and *gestational age in weeks* (22–27, 28–36; 37; 38; 39; 49; 41; 42; 43–46; unknown number) were all coded as dichotomous (dummy coded) and only included in the prediction modeling of smoking prevalence.

### Linear regression with known vs unknown smoking status as outcome

We performed linear multivariable regression with consent vs no-consent (0/1) as outcome, and with the same demographic variables as in the bivariable analyses (Table 1), but with age of mother, parity and birth year as continuous variables. Additional variables included were notification form (electronic or paper form) and institutions. We used a log-risk model, Poisson family (GLM), with the option *vce* (cluster: ID_MOTHER) to account for the same mothers contributing to more than one birth during the study period. We did binomial regressions with relative risks (RRs), assuming somewhat increased standard errors (SEs) compared to log-risk models with *binreg* (binomial family) in STATA. We used STATA version 15 (StataCorp, College Station, Texas).

**Table 1:** SGA-births in percent (95% CI) among mothers with known and unknown smoking status. *N* = 904,982.

	1999–2006			2007–2014		
	Number of births (N)	Known smoking SGA % (CI)	Unknown smoking SGA % (CI)	Number of births (N)	Known smoking SGA % (CI)	Unknown smoking SGA % (CI)
All	436,114	7.4 (7.4–7.5)	8.6 (8.4–8.8)	468,868	8.4 (8.3–8.5)	9.8 (9.5–10.0)
**Age of mother**						
< =19 years	10,487	10.6 (10.0–11.3)	12.9 (11.1–14.8)	9459	11.8 (11.1–12.5)	13.2 (11.3–15.3)
20–29 years	211,688	7.8 (7.7–7.9)	9.1 (8.8–9.5)	215,520	9.0 (8.9–9.1)	10.8 (10.4–11.2)
30–39 years	204,217	6.8 (6.7–6.9)	7.9 (7.6–8.2)	229,046	7.6 (7.5–7.7)	8.9 (8.6–9.2)
> =40 years	9722	8.6 (8.0–9.3)	8.7 (7.4–10.3)	14,843	9.7 (9.2–10.2)	9.8 (8.7–11.0)
**Marital status**						
Married, living together, etc.	402,035	7.2 (7.1–7.3)	8.3 (8.1–8.5)	432,971	8.1 (8.1–8.2)	9.5 (9.3–9.8)
Divorced, living alone, etc.	34,079	10.4 (10.1–10.8)	12.5 (11.6–13.5)	35,897	11.7 (11.3–12.0)	12.4 (11.5–13.3)
**Parity**						
No previous child	177,317	10.1 (10.0–10.3)	11.5 (11.1–11.9)	198,568	11.7 (11.5–11.8)	12.7 (12.4–13.1)
One previous child	155,543	5.6 (5.5–5.8)	6.4 (6.0–6.7)	168,674	6.1 (6.0–6.2)	7.2 (6.9–7.5)
Two + pr. Children	103,254	5.5 (5.4–5.7)	6.7 (6.3–7.1)	101,626	5.9 (5.8–6.1)	7.8 (7.3–8.2)
**Country region of origin**						
Norway/ Nordic countries	383,385	6.9 (6.8–7.0)	7.7 (7.4–7.9)	375,348	7.7 (7.6–7.8)	8.4 (8.2–8.7)
Europe outside the Nordic region	16,013	8.5 (8.0–9.0)	8.9 (7.9–10.0)	37,296	9.0 (8.7–9.3)	9.6 (8.9–10.3)
Asia	23,864	14.2 (13.7–14.7)	15.0 (14.0–16.0)	32,864	13.2 (12.8–13.7)	15.3 (14.5–16.1)
Africa	9321	13.7 (12.9–14.6)	13.9 (12.6–15.3)	17,848	14.7 (14.1–15.3)	15.5 (14.4–16.5)
Others ^a^	3531	8.0 (7.0–9.1)	9.5 (7.3–12.1)	5512	9.9 (9.1–10.8)	10.5 (8.7–12.5)
**Education** ^b^						
University etc.	177,919	6.1 (6.0–6.2)	7.0 (6.7–7.3)	230,175	7.4 (7.3–7.5)	8.5 (8.2–8.8)
Medium	156,437	7.1 (7.0–7.2)	8.2 (7.8–8.6)	125,663	8.0 (7.8–8.2)	9.2 (8.8–9.7)
Primary	82,492	9.8 (9.6–10.1)	10.9 (10.3–11.4)	83,018	10.7 (10.5–10.9)	12.0 (11.4–12.6)

^a^Others: America (North and South) and Oceania

^b^*N* = 432,708 in 1999–2006 and 454,450 in 2007–2014. Missing on education is 4.4% in 1999–2006 and 6.4% in 2007–2014 (1.3% missing among Norwegians/Nordic countries in both periods together)

We used simple linear regression in unadjusted analyses, where the association with each potential independent variable with the outcome could be described as RRs with 95% confidence intervals (CIs).

### Predicting smoking prevalence in the group with unknown smoking status

The machine learning algorithms were modelled using R version 3.6.2. We used an artificial neural networks (*nnet* in the *caret* package) algorithm to build prediction models for maternal smoking prevalence: one for each year, a total of 16 models [15]. A total of 54 dummy variables were included for each model. For each year, we used a subset of 30% of the pregnancies with known smoking prevalence. For each annual subset we increased the weight of the observed prevalence to match the expected smoking prevalence at MNAR. We used 70% of each annual subset as training data to develop the prediction models which were then applied to the remaining 30% as hold-out set for validation of the model performance. The models were trained using a repeated 10-fold cross-validation with 5 repeats. We aimed at developing models to classify the smoking prevalence using the *OptimalCutpoints* package to set the threshold to MNAR smoking prevalence for classification in the training models. For each model we determined the accuracy by predicting the prevalence with 95% confidence intervals in the hold-out set. Finally, we summarized the observed prevalence of smoking for those with known smoking and the predicted prevalence for those with unknown smoking status.

## Results

**Fig. 1:**
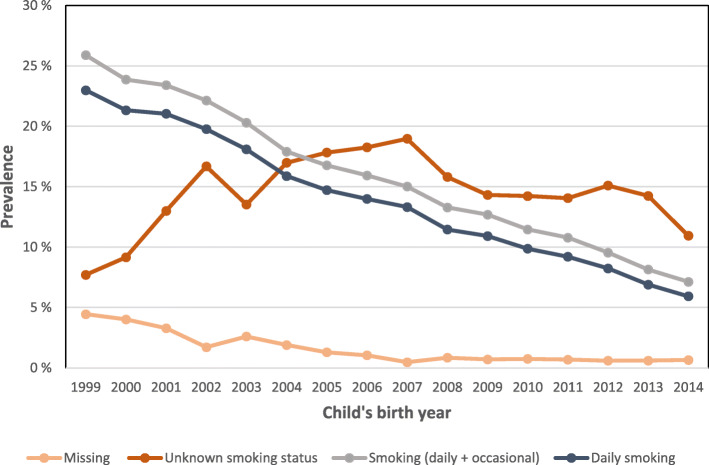
Maternal smoking in first trimester, unknown smoking status and data missing on informed consent. 1999–2014

In our study population of 904,982 births, mean age of the mothers were 29.6 years and 42% were nulliparous. The prevalence of smoking in first trimester fell from 26 to 7% from 1999 to 2014, daily smoking from 23 to 6%. The overall percentage of mothers with unknown smoking status was 14.4%, with the lowest value (8%) in 1999, and the highest in 2007 with 19%. Data missing for the consent-variable was below 5% for all years and below 1% after the year 2006 (Fig. 1).

Mean birth weight of the offspring decreased according to the reported number of daily cigarettes consumed in first trimester. The overall mean birth weight of the offspring of daily smokers was 3428 g. Offspring of occasional smokers had a birth weight significantly lower (3519 g), but close to the birth weight of non-smokers (3536 g). Offspring of mothers with unknown smoking status had a birth weight of 3470 g, falling between that of occasional smokers and low-intensity daily smokers (Supplement S2).

Electronic notification forms were introduced in MBRN in 2005, and rapidly replaced paper-based forms by up to 60% in 2007 and 80% by the year 2009 and increased to nearly 100% by 2014. The proportion of women with unknown smoking status decreased from about one in five in 2007 to one in ten in 2014. A similar low percent of unknown smoking status was also observed in the first years of the study period prior to the introduction of the electronic forms (Fig. 1). In the second period 2007–2014, 18% of all forms were paper based, and unknown smoking status was registered for 42% on the paper forms compared to 9% on the electronic forms.

**Fig. 2:**
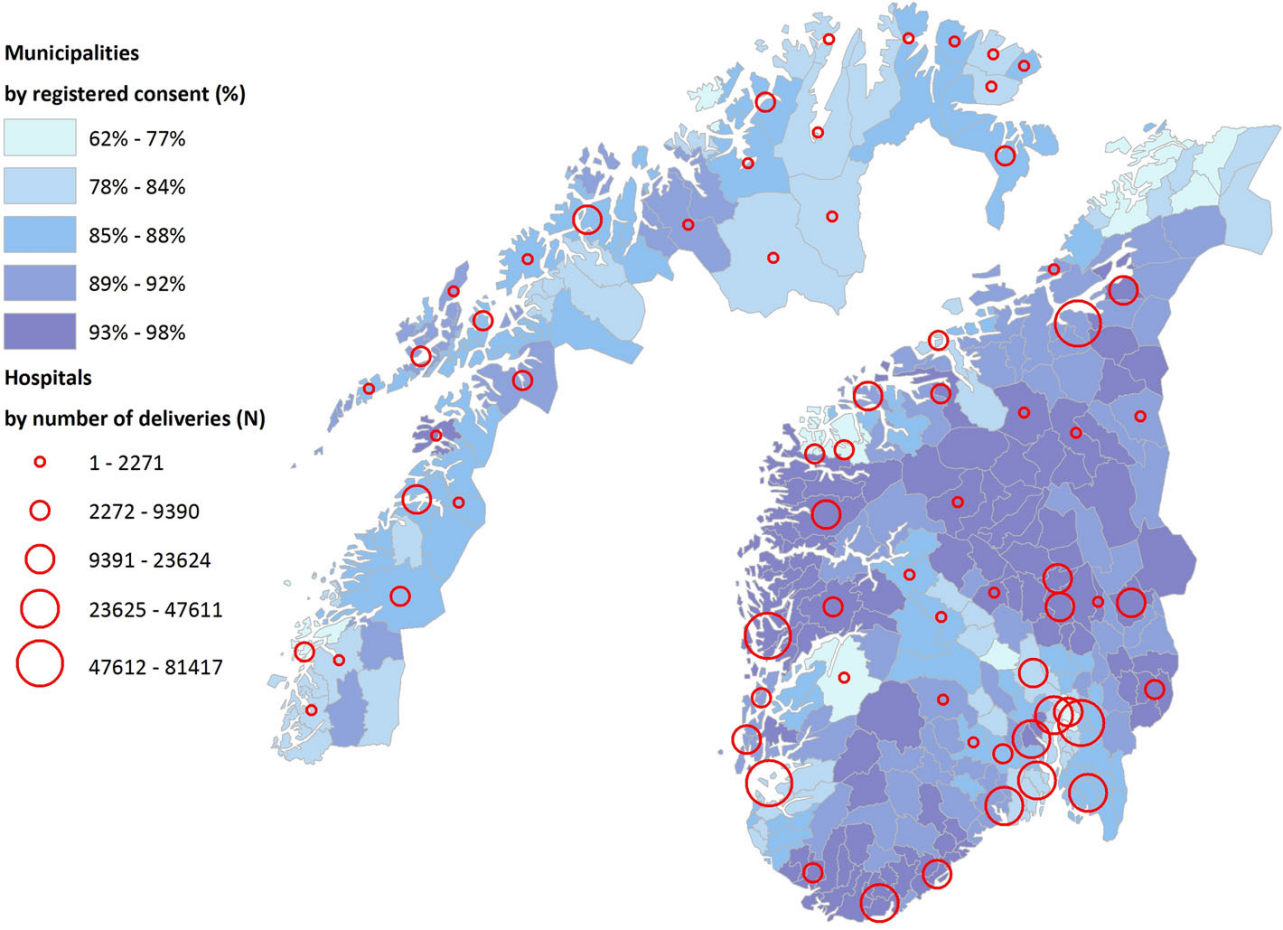
Hospital size and degree of consent among pregnant women by municipality. Norwegian municipalities 2020 (*N* = 356)_a_. _a_Percentage of consent by five classes, hospitals by five classes. The map is our own, using MBRN data from the current study

The prevalence of births with unknown maternal smoking status was slightly lower in the first period: 14.1 (CI 14.0–14.3) than in the second period: 14.7 (CI 14.6–14.8). Women from the Nordic countries had a lower prevalence of unknown smoking status (13.2%) than women from other parts of Europe (17.7%), and women from Africa and Asia (20–30%), Supplement S3. Furthermore, there were geographical differences in the prevalence of known smoking status by residential municipality in Norway ranging from 62 to 98% during the observation period (Fig. 2).

Higher proportions of SGA were found in births with unknown maternal smoking status compared to births with known maternal smoking status, and the proportions of births that were SGA were higher in the last than in the first time period (Table 1). Generally, the highest SGA proportions were found in births to single mothers, mothers younger than 20 years of age, and nulliparous women, regardless of known or unknown smoking status. SGA births were also more prevalent among women who were born outside the Nordic region. Pronounced gradients in the proportion of SGA-births by educational level were found in both groups of known and unknown smoking status, with the highest levels of SGA-births among women with primary education (less than 11 school years) (Table 1).

### Factors associated with unknown smoking status

In the multivariable regression analyses, mothers’ age showed no association, while parity was positively associated with unknown smoking status (Table 2). Education was positively, although relatively weakly, associated with known smoking status. The adjusted increased risk to have unknown smoking status was 2% among women with SGA-births (non-SGA-births as reference). A stronger association was found for maternal country region of origin. Compared to women from Nordic countries, women from non-Nordic European countries had 15% higher risk of having an unknown smoking status (RR 1.15), while Asian and the African women had 17 and 26% (RR 1.17 and 1.26) higher risk, respectively. Electronic notification forms, mainly introduced in the last time period (2007–2014), were associated with 42% lower prevalence of unknown smoking status relative to paper-based notifications (RR 0.58; CI 0.57–0.59). There were large differences between hospitals, ranging from RR 4.74 and RR 2.43 for having an unknown smoking status in two hospitals in the Oslo region to RR 0.33 in a hospital in the middle eastern region of Norway relative to our reference group of hospitals with less than 20,000 births for all years of the study period (Table 2). In a sensitivity analysis where only electronic notification forms were included (*N* = 360,351), we found more pronounced differences regarding country region of origin and education, but less differences between the hospitals, compared to the model in Table 2 (data not shown).

**Table 2:** Regression analyses. Factors associated with unknown smoking status. *N* = 855,704.

Known smoking status = 0 / Unknown smoking status = 1	1999–2014	
	Unadjusted	Full model ^a^
	RR (95% CI)	RR (95% CI)
Continuous variables:		
Age of mother	1.02 (1.01–1.02)	1.00 (1.00–1.00)
Parity	0.98 (0.98–0.99)	1.02 (1.02–1.03)
Year of child birth	1.01 (1.01–1.01)	1.03 (1.03–1.03)
Paper-based form	ref	ref
Electronic form	0.47 (0.47–0.48)	0.58 (0.57–0.59)
Norway/ Nordic Countries	ref	ref
Europe outside the Nordic region	1.35 (1.32–1.38)	1.15 (1.12–1.17)
Asia	1.68 (1.65–1.71)	1.17 (1.15–1.19)
Africa	2.02 (1.98–2.07)	1.26 (1.23–1.29)
Others	1.42 (1.35–1.49)	1.09 (1.04–1.14)
No SGA-10	ref	ref
SGA-10	1.15 (1.13–1.17)	1.02 (1.00–1.04)
Married, living together, etc.	ref	ref
Divorced, living alone, etc.	1.01 (1.00–1.03)	1.02 (1.00–1.04)
Medium education	ref	ref
University education	1.19 (1.18–1.21)	0.97 (0.96–0.98)
Primary education	1.13 (1.12–1.15)	1.04 (1.02–1.06)

^a^Full model, with institutions included. Compared to the reference group “small hospitals”, the RR of the 16 largest hospitals ranged from RR 0.33 (CI 0.30–0.36) to RR 4.74 (CI 4.67–4.83)

### Machine learning prediction of smoking prevalence among mothers with unknown smoking status

For each year we calculated the ratio of the SGA prevalence in the unknown group to the SGA prevalence in the known group, as smoking may be seen as an important reason for MNAR. This ratio was then multiplied with the observed smoking prevalence (Supplement S4, Table A and Figure A). For instance, the observed smoking prevalence was 25.6% in 1999, the SGA-ratio (unknown smoking status/known smoking status) was 1.06, where the MNAR smoking prevalence was set to 25.6%*1.06 = 27.2%. The ratio of SGA for the groups of unknown and known smoking status gave the assumption of a higher smoking prevalence in the range of 6–24% in the group of unknown smoking status, and this was used to scale the observed smoking prevalence.

The number of observations included in the model-building (training) ranged from 13,261 (2002) to 15,504 (2009). The overall accuracy of the models was in the range from 0.970 through 1.000. The predicted smoking prevalence in the hold-out data was in the range of the observed values except for 2011 (Supplement S4, Table B). For this year, the model underestimated the prevalence on average by 0.3 percentage points (i.e., 2.1%). Overall, the most important predictor variables were education, ethnic background, marital status and birth weight (Supplement S4, Figure B).

**Fig. 3:**
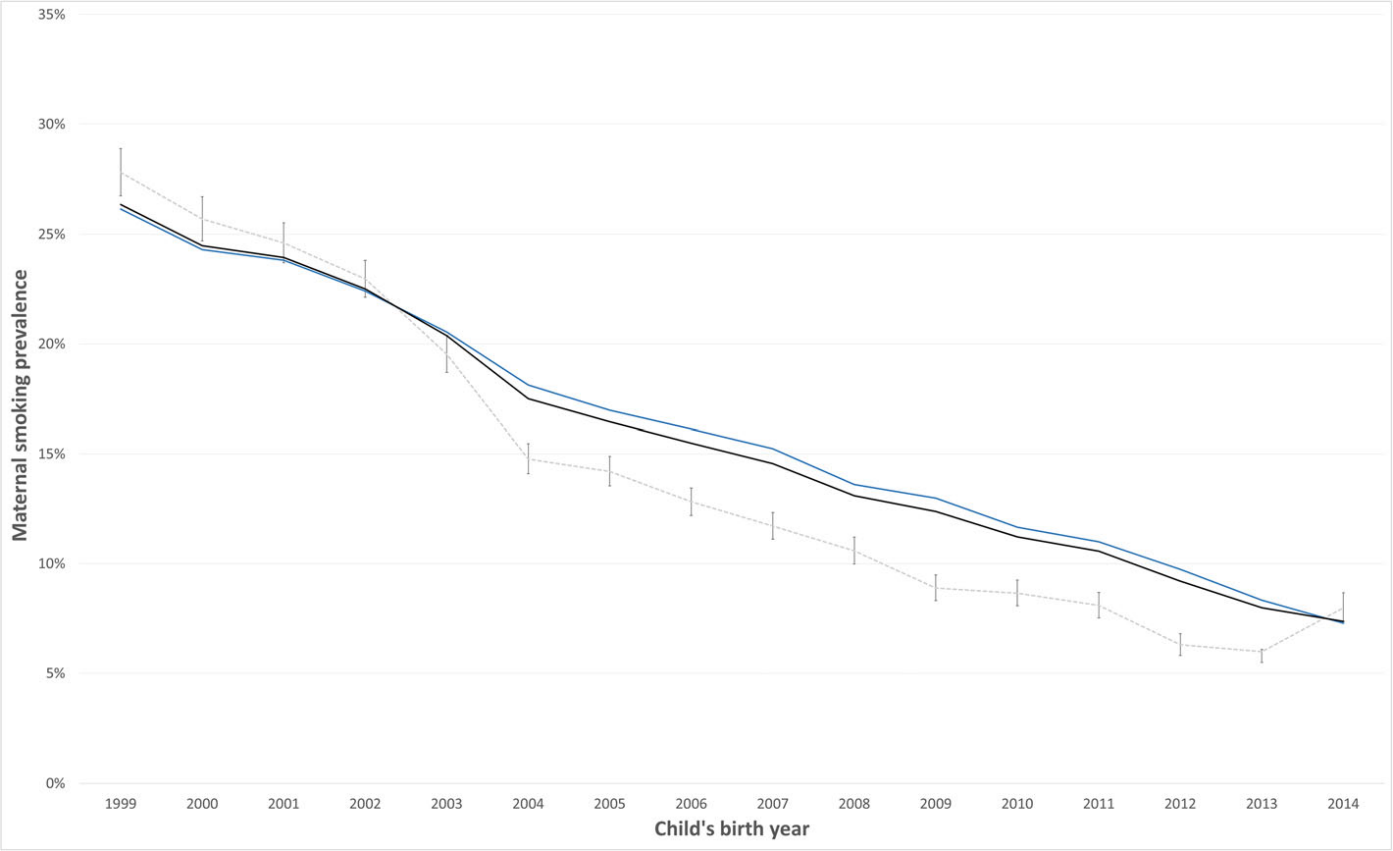
Observed prevalence (blue line), MNAR predicted prevalence (dotted grey line), and combined prevalence (black line)_a_. _a_Prevalence of maternal smoking pro year. Percentage and 95% CIs for predicted prevalence in the non-consent group

We used the annual models to classify maternal smoking (non-smoker/smoker) in the data without consent. The predicted prevalence was overall lower than the prevalence observed among cases with consent (Supplement S4, Table C). Fig. 3 shows the combined prevalence (observed + predicted) with an overall reduction in smoking in the range of 0.8% (2003) to 5.5% (2012), with an exception of a slight increase of 0.4–0.8% before 2003 and 1.1% in 2014.

Due to the potential of unmeasured confounding from maternal BMI, a sensitivity analysis was performed including this variable for the available years (2007–2014, *N* = 215,535). In addition, we performed a sensitivity analyses to examine possible intermediate confounding from maternal education (*N* = 904,982). These tests did not show any substantial difference from the main model that would change the interpretation of Fig. 3 (data not shown).

## Discussion

Despite the known problem of missing smoking data from birth registrations in numerous studies, few studies have evaluated the extent of bias that may be associated with unknown or incomplete smoking data among pregnant women. In the current study, we utilized traditional and a machine learning strategy to investigate if incomplete data on smoking status in MBRN may have biased the reported smoking prevalence in the first trimester of pregnancy and the trends over a 16-year time period.

The characteristics most related to unknown maternal smoking status included foreign country of origin, paper-based registration forms as opposed to electronic forms, and selected hospitals. The machine learning exercise which predicted smoking status among those not providing smoking information resulted in an overall combined (observed plus predicted) trend in the prevalence of smoking that was similar to the trend in the observed prevalence of smoking. Thus, despite the high percent missing smoking data (i.e., as high as 18% for some years), the known smoking status in the MBRN likely provides reliable estimates and trends for the general population of pregnant women.

### Traditional approach

We found higher levels of unknown smoking status among women from non-Nordic countries. The extent of incomplete smoking data may reflect cultural differences and language barriers between hospital staff and mothers. The higher rates of unknown smoking status in some hospitals may be due to geographical differences in the proportion of immigrants, large number of births with busy staff and other local differences in the registrations, as well as prolonged use of paper-based notification forms. Electronic forms were associated with lower rates of non-consent among all pregnant women, independent of country region of origin. The factors contributing to unknown smoking status may be diverse, with the active choice “to refrain from providing smoking information” as one of several possible reasons for incomplete smoking data.

We found slightly elevated prevalence of SGA among women with unknown smoking status, possibly indicating a higher prevalence of smoking in this group than among women with known smoking status. However, the high proportion of women from non-Nordic country regions with unknown smoking status may reflect, to some degree, a higher prevalence of SGA even in the presence of a lower smoking prevalence [2].

In our study, women with university education had a higher tendency to consent to provide smoking information than women with primary education. Our study investigates possible reasons for unknown smoking status, and may not be directly comparable with underreporting smoking among women with available smoking information. However, studies from other countries suggest that there is greater underreporting of maternal smoking among women with high than with low socioeconomic status. A study from six states in the US found that the completeness of ascertainment of prenatal smoking decreased as women’s education increased [12]. In Scotland a greater proportion of the smokers in the least deprived areas did not report their smoking (39%) than women in the most deprived areas (22%). However, smoking was far more common in the most deprived areas than in affluent areas [16].

### Machine learning approach

The machine learning approach found that education level and country of origin was strong predictors for smoking prevalence, in addition to birth weight and marital status. All other factors considered in the prediction analyses, assuming similar associations between the predictor variables and smoking in both groups of known and unknown smoking status at MNAR, the overall predicted smoking prevalence in women with unknown smoking status was lower than the observed prevalence in women with known smoking status. Fig. 3 depicts greater differences between the predicted smoking prevalence in the “unknown-group” than the observed prevalence in the “known-group” in the later years of the study period. This may have been influenced by the increasing proportion of women with low smoking prevalence from other country regions of origin in the “unknown-group”.

The increase in the predicted smoking prevalence in the last year, coincide with nearly 100% electronic registrations achieved in 2014, and may have excluded some of the irrelevant reasons for non-consent, thus making the smokers in the unknown-group more visible. In addition, an increasing use of snus among young women in Norway, which is not yet available in the MBRN, may have influenced the SGA ratio used in the scaling of the prediction models in the latest years of our observation period [17, 18].

### Incomplete vs underreported smoking data

In Norway, the mother’s informed consent for collecting smoking data is required, in contrast to policies of birth registers in the other Nordic countries [19]. A study from six US states found low completeness of prenatal smoking ascertainment in birth certificates and outlined the many data collection steps that could contribute to inaccuracies in reporting [12]. The prenatal care provider asks the pregnant woman about her smoking status, then the provider records the smoking status in the medical record, then the medical record is sent to the birth hospital, then the medical administrative staff records the smoking status on the birth certificate work sheet [12]. The way from the “Health card for pregnant women” at the first antenatal check-up in primary care, via the hospitals births records, to the MBRN, where also informed consent is required, is certainly not less complicated. In a recent study about hyperactivity disorder and maternal smoking in Norway, almost one of five deliveries were excluded because of missing or incomplete smoking information [6].

Few studies are found on unknown or incomplete smoking data among pregnant women. In a study from the Swedish MBR (MBRS), 6.5% of births were missing maternal smoking status [20], but without further information about the reasons for missing. Another large study used birth registry data from Sweden and Norway in evaluating the incidence of malformations associated with pregnancy exposure to the drug modafinil. Interestingly, smoking information was missing in 8% of the unexposed but only in 4.5% of the exposed mothers in the study [21]. Mothers exposed to the drug had higher levels of smoking, comorbidities and other medication use than the unexposed women. The greater degree of smoking ascertainment in the modafinil drug users than non-users may reflect more time and interest given both in the antenatal care and from the hospital staff in the birth clinics, compared to the unexposed [21].

A study on self-reported and cotinine-validated smoking status in Estonia [22], reported a cotinine-adjusted smoking prevalence of 24.6% among women that reported their smoking status during pregnancy (*N* = 1333). A small group of women not reporting their smoking status (*N* = 27), had a cotinine-adjusted smoking prevalence of 22.2%. These results are comparable to those from a study from Scotland [16], where the cotinine-adjusted smoking prevalence was 30.5% among women that reported their smoking status during pregnancy (*N* = 3177) and 26.2% among women that did not report smoking status (*N* = 298). These studies are in line with our predicted smoking prevalence among the mothers without smoking status.

Although not the main focus of this study, the possibility of underreporting of smoking in MBRN should be considered. In studies where smoking prevalence is based on self-report, evidence of underestimation is found where the degree of bias depends upon the population examined. Pregnant women are particularly prone to underreporting of smoking, as smoking in pregnancy is seen as socially undesirable [14, 23], perhaps also due to concern about the child and may reflect a desire or decision to quit smoking during pregnancy. However, validation studies from MBRS and from the Norwegian Mother and Child Cohort Study have found low levels of underreporting [24, 25]. In contrast, higher rates of underreporting of maternal smoking in validation studies based upon serum or urinary cotinine have been described in studies from New Zealand and USA [9, 23].

### Strengths and limitations

Major strengths of our study include the population-based study design of a nation-wide cohort of more than 900,000 pregnant women. The prospective collection of smoking status would minimalize recall bias, and a high agreement between self-reported smoking in pregnancy and the mothers’ levels of serum cotinine has been found in MBRS and in other validated studies in Scandinavia [24–26]. Another strength in MBRN is a clear dose-response association between number of cigarettes smoked daily in first trimester and birth weight of the offspring. Altogether, this suggests mainly accurate reporting among those consenting to provide smoking information in the MBRN.

Environmental tobacco smoke exposure is not registered in the MBRN, and has been found difficult to identify without biomarker assessments [26]. As the prevalence of maternal smoking declined, the probability of underreporting smoking may have increased in recent years, given the relationship between reporting bias and social desirability, and the decline in social acceptability of smoking [14]. In the last decade, the smoking prevalence from MBRN does not give a complete picture of overall tobacco use among pregnant women, as smokeless tobacco (snus) has gained increasing popularity [27]. National data on snus use was not available in our study.

## Conclusion

Despite missing smoking data due to non-consent to providing smoking information in MBRN, our assessment identified total estimates of smoking that were very similar to the smoking prevalence among the women with known smoking status. Under the assumption of similar associations between our predictors and smoking in both groups of known and unknown smoking status, our analyses have substantiated that for most purposes it will be acceptable to use the observed smoking information without introducing notable bias. However, even when the number of women with unknown smoking status has decreased in the latest years, a continuous effort to obtain a most complete registration of smoking information would positively affect the validity of the MBRN-data.

## Supplementary Information

**Additional file 1: Supplement S1.** Flow chart.

**Additional file 2: Supplement S2.** Difference from mean birth weight for children of known non-smoking mothers (g). (Bar chart with supporting table).

**Additional file 3: Supplement S3.** Prevalence of unknown smoking status (non-consent), in percent (95% CI). *N*= 904,982.

**Additional file 4: Supplement S4.** Validation. (Supporting information in tables and figures to the machine learning prediction analyses).

## Data Availability

Data from MBRN may be applied for at the Norwegian Institute of Public Health. The data in the current study are linked to population data from Statistics Norway, and restrictions apply to the availability of the data, according to the license from the Regional Committee on Medical and Health Research Ethics. Anonymized data are however available from the authors upon reasonable request.

## Abbreviations

MBRN: Medical Birth Register of Norway
MBRS: Medical Birth Register of Sweden
RR: Relative Risk
SE: Standard Error
CI: Confidence Interval
SGA: Small for Gestation Age
vs: Versus
MNAR: Missing-not-at-random
MAR: Missing-at-random
