# Supplementary figures and images for: Evaluation of incomplete maternal smoking data using machine learning algorithms: a study from the Medical Birth Registry of Norway
Source: BMC Pregnancy Childbirth. 2020 Nov 23;20:710. doi: 10.1186/s12884-020-03384-y (PMC7684740; doi:10.1186/s12884-020-03384-y)

# Supplement S1

Medical Birth Registry Norway  
All births 1999-2014  
N = 960 408  
Age of mothers 13-59

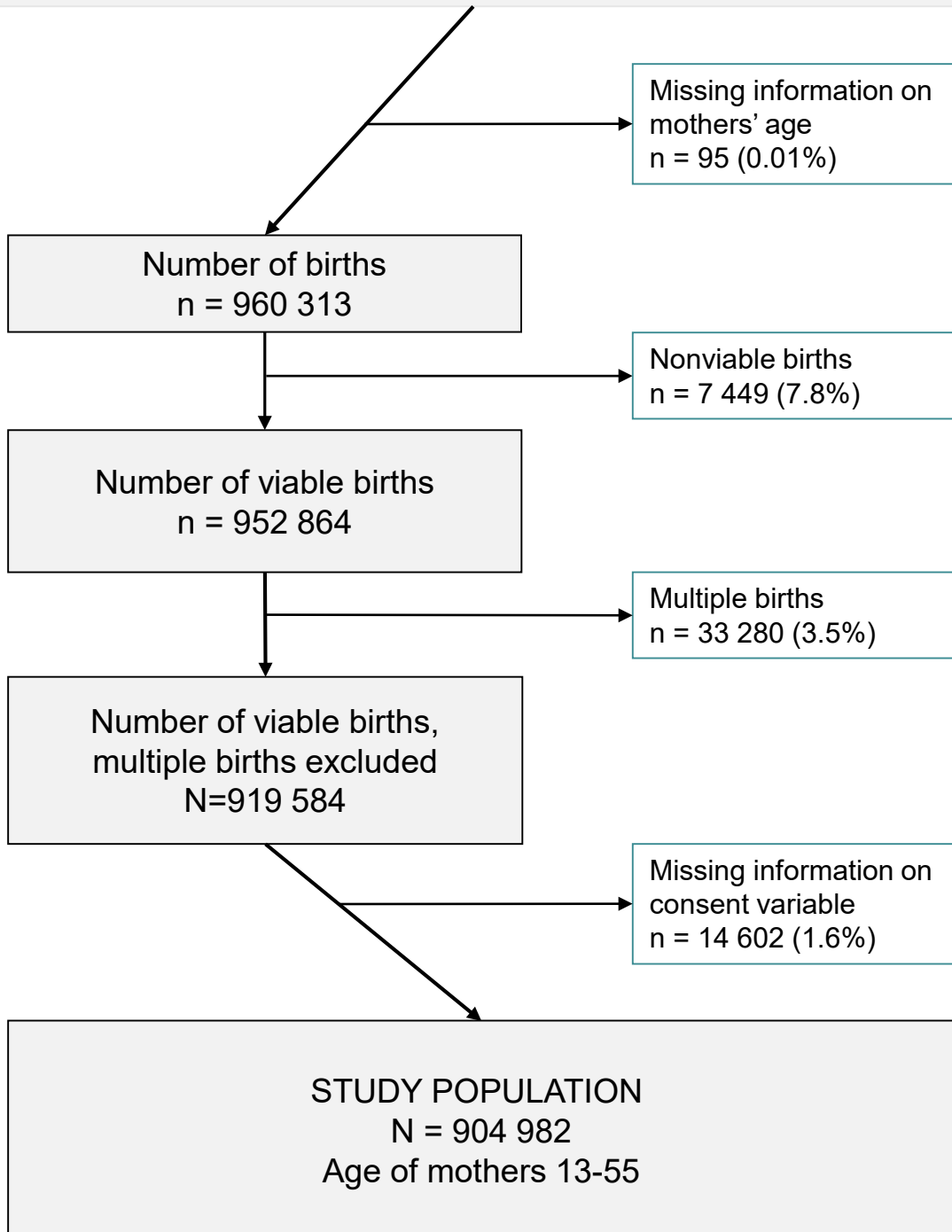

Supplement: Supplementary file 1 — Additional file 1: Supplement S1. Flow chart. [file 12884_2020_3384_MOESM1_ESM.pdf]
