# Supplementary material for: Evaluation of incomplete maternal smoking data using machine learning algorithms: a study from the Medical Birth Registry of Norway
Source: BMC Pregnancy Childbirth. 2020 Nov 23;20:710. doi: 10.1186/s12884-020-03384-y (PMC7684740; doi:10.1186/s12884-020-03384-y)
Supplement: Supplementary file 2 — Additional file 2: Supplement S2. Difference from mean birth weight for children of known non-smoking mothers (g). (Bar chart with supporting table). [file 12884_2020_3384_MOESM2_ESM.docx]

Supplement S2. Difference from mean birth weight for children of known non-smoking mothers (g).

|  |
| --- |

| Number of cigarettes per day | Number | Mean birth weight | SD | Difference |
| --- | --- | --- | --- | --- |
| Non-smoker | 670 098 | 3 536 | 601 | 0 |
| 1-5 cigarettes | 42 303 | 3 437 | 614 | 99 |
| 6-10 cigarettes | 40 290 | 3 380 | 620 | 156 |
| 11-15 cigarettes | 12 519 | 3 357 | 621 | 179 |
| 16-20 cigarettes | 7 186 | 3 350 | 645 | 186 |
| 20+ cigarettes | 1 043 | 3 300 | 658 | 236 |
| Occasional smoking | 15 079 | 3 519 | 611 | 17 |
| Daily smoking, unknown amount | 6 049 | 3 408 | 649 | 128 |
| Unknown smoking status | 150 928 | 3 470 | 627 | 66 |
