# Supplementary material for: Evaluation of incomplete maternal smoking data using machine learning algorithms: a study from the Medical Birth Registry of Norway
Source: BMC Pregnancy Childbirth. 2020 Nov 23;20:710. doi: 10.1186/s12884-020-03384-y (PMC7684740; doi:10.1186/s12884-020-03384-y)
Supplement: Supplementary file 3 — Additional file 3: Supplement S3. Prevalence of unknown smoking status (non-consent), in percent (95% CI). N= 904,982. [file 12884_2020_3384_MOESM3_ESM.docx]

Supplement S3. Prevalence of unknown smoking status (non-consent), in percent (95% CI). N= 904,982.

|  | 1999-2006 | | 2007-2014 | |
| --- | --- | --- | --- | --- |
|  | **Number of births N** | **Non-consent % (CI)** | **Number of births N** | **Non-consent % (CI)** |
| All | 436 114 | 14.1 (14.0-14.3) | 468 868 | 14.7 (14.6-14.8) |
| Age of mother |  |  |  |  |
| <=19 years | 10 487 | 12.8 (12.1-13.4) | 9 459 | 11.9 (11.3-12.6) |
| 20-29 years | 211 688 | 13.6 (13.4-13.7) | 215 520 | 13.3 (13.1-13.4) |
| 30-39 years | 204 217 | 14.8 (14.6-14.9) | 229 046 | 16.0 (15.9-16.2) |
| >=40 years | 9 722 | 15.8 (15.1-16.5) | 14 843 | 17.2 (16.6-17.8) |
|  |  |  |  |  |
| Marital status |  |  |  |  |
| Married, living together, etc. | 402 035 | 14.2 (14.1-14.3) | 432 971 | 14.6 (14.5-14.7) |
| Divorced, living alone, etc. | 34 079 | 13.7 (13.3-14.0) | 35 897 | 15.6 (15.2-15.9) |
|  |  |  |  |  |
| Parity |  |  |  |  |
| No previous child | 177 317 | 14.7 (14.6-14.9) | 198 568 | 15.3 (15.2-15.5) |
| One previous child | 155 543 | 13.8 (13.6-14.0) | 168 674 | 14.4 (14.3-14.6) |
| Two + pr. children | 103 254 | 13.7 (13.5-13.9) | 101 626 | 13.9 (13.7-14.1) |
|  |  |  |  |  |
| Country region of origin |  |  |  |  |
| Norway/ Nordic countries | 383 385 | 13.1 (13.0-13.3) | 375 348 | 13.2 (13.1-13.3) |
| Europe outside the Nordic region | 16 013 | 18.7 (18.1-19.3) | 37 296 | 17.2 (16.8-17.6) |
| Africa | 9 321 | 27.1 (26.2-28.0) | 17 848 | 26.2 (25.5-26.8) |
| Asia | 23 864 | 21.6 (21.0-22.1) | 32 864 | 22.2 (21.8-22.7) |
| Others _a_ | 3 531 | 17.6 (16.3-18.9) | 5 512 | 19.2 (18.2-20.3) |
|  |  |  |  |  |
| Education _b_ |  |  |  |  |
| University etc. | 177 919 | 14.7 (14.5-14.8) | 230 175 | 15.1 (15.0-15.3) |
| Medium | 156 437 | 12.5 (12.3-12.7) | 125 663 | 12.5 (12.3-12.7) |
| Primary | 82 492 | 14.1 (13.8-14.3) | 83 018 | 14.3 (14.0-14.5) |

_a_ Others: America (North and South) and Oceania

_b_ N= 436,114 in 1999-2006 and 468,868 in 2007-2014. Missing on education was 4.4% in 1999-2006 and 6.4% in 2007-2014 (1.3% missing among Norwegians/Nordic countries in both periods together).
