# Supplementary material for: Evaluation of incomplete maternal smoking data using machine learning algorithms: a study from the Medical Birth Registry of Norway
Source: BMC Pregnancy Childbirth. 2020 Nov 23;20:710. doi: 10.1186/s12884-020-03384-y (PMC7684740; doi:10.1186/s12884-020-03384-y)
Supplement: Supplementary file 4 — Additional file 4: Supplement S4. Validation. (Supporting information in tables and figures to the machine learning prediction analyses). [file 12884_2020_3384_MOESM4_ESM.docx]

Supplement S4 Validation

TABLE A. Prevalence of SGA by consent, SGA-ratio, smoking prevalence observed and MNAR-predicted. MBRN-data 1999-2014.

|  | **SGA (%)** | |  | **Smoking prevalence (%)** | |
| --- | --- | --- | --- | --- | --- |
| **Year** | **Consent group** | **Non-consent group** | **SGA-ratio** | **Observed** | **MNAR** |
|  |  |  |  |  |  |
| 1999 | 7,6 % | 8,0 % | 1,064 | 25,6 % | 27,2 % |
| 2000 | 7,3 % | 7,9 % | 1,080 | 23,8 % | 25,7 % |
| 2001 | 7,4 % | 8,3 % | 1,111 | 23,3 % | 25,9 % |
| 2002 | 7,2 % | 9,0 % | 1,241 | 22,0 % | 27,4 % |
| 2003 | 7,3 % | 8,7 % | 1,196 | 20,3 % | 24,2 % |
| 2004 | 7,1 % | 8,6 % | 1,209 | 17,9 % | 21,6 % |
| 2005 | 7,7 % | 8,8 % | 1,143 | 16,8 % | 19,2 % |
| 2006 | 7,9 % | 8,9 % | 1,129 | 15,9 % | 18,0 % |
| 2007 | 8,3 % | 9,0 % | 1,089 | 15,0 % | 16,3 % |
| 2008 | 8,2 % | 9,9 % | 1,209 | 13,3 % | 16,0 % |
| 2009 | 8,3 % | 9,5 % | 1,142 | 12,7 % | 14,5 % |
| 2010 | 8,7 % | 9,5 % | 1,088 | 11,5 % | 12,5 % |
| 2011 | 8,7 % | 10,2 % | 1,174 | 10,8 % | 12,7 % |
| 2012 | 8,4 % | 9,6 % | 1,143 | 9,5 % | 10,9 % |
| 2013 | 8,4 % | 10,2 % | 1,216 | 8,1 % | 9,9 % |
| 2014 | 8,3 % | 10,1 % | 1,210 | 7,1 % | 8,6 % |

FIGURE A. Observed (blue line) and MNAR-predicted prevalence (dotted grey line) of maternal smoking by year in validation data. Percentage and 95% confidence intervals for predicted prevalence.

TABLE B. Model performance on hold-out validation data.

|  |  | **Testdata** |  | **95% CI** | |  | **95% CI** | |  | **95% CI** | |
| --- | --- | --- | --- | --- | --- | --- | --- | --- | --- | --- | --- |
| **Year** | **Count** | **Observed** | **Predicted** | **lower** | **upper** | **Accuracy** | **lower** | **upper** | **Error** | **lower** | **upper** |
|  |  |  |  |  |  |  |  |  |  |  |  |
| 1999 | 35 443 | 0,272 | 0,279 | 0,263 | 0,295 | 1,007 | 0,991 | 0,977 | 0,007 | 0,009 | 0,023 |
| 2000 | 34 998 | 0,257 | 0,254 | 0,238 | 0,271 | 0,997 | 0,981 | 1,014 | 0,003 | 0,019 | 0,014 |
| 2001 | 32 387 | 0,259 | 0,259 | 0,241 | 0,277 | 1,000 | 0,982 | 1,018 | 0,000 | 0,018 | 0,018 |
| 2002 | 30 941 | 0,274 | 0,272 | 0,253 | 0,291 | 0,998 | 0,979 | 1,017 | 0,002 | 0,021 | 0,017 |
| 2003 | 32 524 | 0,242 | 0,240 | 0,222 | 0,258 | 0,998 | 0,980 | 1,016 | 0,002 | 0,020 | 0,016 |
| 2004 | 31 656 | 0,216 | 0,203 | 0,186 | 0,220 | 0,986 | 0,970 | 1,004 | 0,014 | 0,030 | 0,004 |
| 2005 | 31 380 | 0,192 | 0,191 | 0,175 | 0,207 | 0,999 | 0,983 | 1,015 | 0,001 | 0,017 | 0,015 |
| 2006 | 32 338 | 0,180 | 0,176 | 0,161 | 0,192 | 0,996 | 0,981 | 1,012 | 0,004 | 0,019 | 0,012 |
| 2007 | 32 151 | 0,163 | 0,159 | 0,145 | 0,173 | 0,996 | 0,982 | 1,010 | 0,004 | 0,018 | 0,010 |
| 2008 | 34 576 | 0,160 | 0,161 | 0,146 | 0,176 | 1,001 | 0,986 | 1,016 | 0,001 | 0,014 | 0,016 |
| 2009 | 36 173 | 0,145 | 0,137 | 0,124 | 0,150 | 0,992 | 0,979 | 1,005 | 0,008 | 0,021 | 0,005 |
| 2010 | 36 027 | 0,125 | 0,123 | 0,111 | 0,135 | 0,998 | 0,986 | 1,010 | 0,002 | 0,014 | 0,010 |
| 2011 | 35 409 | 0,127 | 0,112 | 0,100 | 0,124 | 0,985 | 0,973 | 0,997 | 0,015 | 0,027 | 0,003 |
| 2012 | 34 989 | 0,109 | 0,104 | 0,092 | 0,116 | 0,995 | 0,983 | 1,007 | 0,005 | 0,017 | 0,007 |
| 2013 | 34 572 | 0,099 | 0,103 | 0,091 | 0,116 | 1,004 | 0,992 | 0,983 | 0,004 | 0,008 | 0,017 |
| 2014 | 35 882 | 0,086 | 0,080 | 0,071 | 0,091 | 0,994 | 0,984 | 1,005 | 0,006 | 0,016 | 0,005 |

FIGURE B. Overall variable importance in the prediction models (1999-2014). Percentage.

TABLE C. Observed and MNAR-predicted maternal smoking prevalence on MBRN-data 1999-2014.

|  | **MNAR - Observed+predicted** | | | | **Observed** | | **Predicted - MNAR** | | | |
| --- | --- | --- | --- | --- | --- | --- | --- | --- | --- | --- |
| **Year** | **Count** | **Prevalence** | **95% lower** | **95% upper** | **Count** | **Prevalence** | **Count** | **Predicted** | **95% lower** | **95% upper** |
|  |  |  |  |  |  |  |  |  |  |  |
| 1999 | 57 413 | 0,263 | 0,262 | 0,265 | 50 633 | 0,262 | 6 780 | 0,278 | 0,268 | 0,289 |
| 2000 | 57 344 | 0,245 | 0,243 | 0,246 | 49 997 | 0,243 | 7 347 | 0,257 | 0,247 | 0,267 |
| 2001 | 54 988 | 0,239 | 0,238 | 0,241 | 46 267 | 0,238 | 8 721 | 0,246 | 0,237 | 0,255 |
| 2002 | 53 990 | 0,225 | 0,224 | 0,227 | 44 202 | 0,224 | 9 788 | 0,230 | 0,221 | 0,238 |
| 2003 | 55 159 | 0,204 | 0,202 | 0,205 | 46 463 | 0,205 | 8 696 | 0,195 | 0,187 | 0,204 |
| 2004 | 55 535 | 0,175 | 0,174 | 0,176 | 45 223 | 0,181 | 10 312 | 0,148 | 0,141 | 0,155 |
| 2005 | 55 261 | 0,165 | 0,163 | 0,166 | 44 828 | 0,170 | 10 433 | 0,142 | 0,135 | 0,149 |
| 2006 | 57 111 | 0,155 | 0,154 | 0,156 | 46 198 | 0,161 | 10 913 | 0,128 | 0,122 | 0,135 |
| 2007 | 56 954 | 0,146 | 0,144 | 0,147 | 45 929 | 0,152 | 11 025 | 0,117 | 0,111 | 0,123 |
| 2008 | 59 173 | 0,131 | 0,130 | 0,132 | 49 394 | 0,136 | 9 779 | 0,106 | 0,100 | 0,112 |
| 2009 | 60 740 | 0,124 | 0,123 | 0,125 | 51 677 | 0,130 | 9 063 | 0,089 | 0,083 | 0,095 |
| 2010 | 60 455 | 0,112 | 0,111 | 0,113 | 51 468 | 0,117 | 8 987 | 0,086 | 0,081 | 0,092 |
| 2011 | 59 274 | 0,106 | 0,105 | 0,107 | 50 585 | 0,110 | 8 689 | 0,081 | 0,075 | 0,087 |
| 2012 | 59 229 | 0,092 | 0,091 | 0,093 | 49 984 | 0,097 | 9 245 | 0,063 | 0,058 | 0,068 |
| 2013 | 57 945 | 0,080 | 0,079 | 0,080 | 49 389 | 0,083 | 8 556 | 0,060 | 0,055 | 0,061 |
| 2014 | 57 944 | 0,074 | 0,073 | 0,074 | 51 260 | 0,073 | 6 684 | 0,080 | 0,074 | 0,087 |
